# Maternal Exposure to Aeroallergens and the Risk of Early Delivery

**DOI:** 10.1097/EDE.0000000000000573

**Published:** 2016-11-30

**Authors:** Eric Lavigne, Antonio Gasparrini, David M. Stieb, Hong Chen, Abdool S. Yasseen, Eric Crighton, Teresa To, Scott Weichenthal, Paul J. Villeneuve, Sabit Cakmak, Frances Coates, Mark Walker

**Affiliations:** From the a Air Health Science Division, Health Canada, Ottawa, Ontario, Canada; b School of Epidemiology, Public Health and Preventive Medicine, University of Ottawa, Ottawa, Ontario, Canada; c Department of Social and Environmental Health Research, London School of Hygiene & Tropical Medicine, London, United Kingdom; d Population Studies Division, Health Canada, Canada; e Public Health Ontario, Toronto, Ontario, Canada; f Dalla Lana School of Public Health, University of Toronto, Toronto, Ontario, Canada; g Institute for Clinical Evaluative Sciences, Ontario, Canada; h Ottawa Hospital Research Institute, Ottawa, Ontario, Canada; i Better Outcomes Registry and Network Ontario, Ottawa, Ontario, Canada; j Children’s Hospital of Eastern Ontario Research Institute, Ottawa, Ontario, Canada; k Department of Geography, University of Ottawa, Ottawa, Ontario, Canada; l Child Health Evaluative Sciences, The Hospital for Sick Children, Toronto, Ontario, Canada; m Department of Epidemiology, Biostatistics and Occupational Health, McGill University, Montreal, Quebec, Canada; n CHAIM Research Centre, Carleton University, Ottawa, Ontario, Canada; and o Aerobiology Research Laboratories, Ottawa, Ontario, Canada; and p Department of Obstetrics and Gynecology, University of Ottawa, Ottawa, ON, Canada.

## Abstract

**Background:**

Daily changes in aeroallergens during pregnancy could trigger early labor, but few investigations have evaluated this issue. This study aimed to investigate the association between exposure to aeroallergens during the week preceding birth and the risk of early delivery among preterm and term pregnancies.

**Methods:**

We identified data on 225,234 singleton births that occurred in six large cities in the province of Ontario, Canada, from 2004 to 2011 (April to October) from a birth registry. We obtained daily counts of pollen grains and fungal spores from fixed-site monitoring stations in each city and assigned them to pregnancy period of each birth. Associations between exposure to aeroallergens in the preceding week and risk of delivery among preterm (<37 gestational weeks), early-term (37–38 weeks), and full-term (≥39 weeks) pregnancies were evaluated with Cox regression models, adjusting for maternal characteristics, meteorologic parameters, and air pollution concentrations, and pooled across the six cities.

**Results:**

The risk of delivery increased by 3% per interquartile range width (IQR_*w*_ = 22.1 grains/m^3^) increase in weed pollen the day before birth among early-term (hazard ratio [HR] = 1.03; 95% confidence interval [CI]: 1.01, 1.05) and full-term pregnancies (HR = 1.03; 95% CI: 1.01, 1.04). Exposure to fungal spores cumulated over 0 to 2 lagged days was associated with increased risk of delivery among full-term pregnancies only (HR = 1.07; 95% CI: 1.01, 1.12). We observed no associations among preterm deliveries.

**Conclusions:**

Increasing concentrations of ambient weed pollen and fungal spores may be associated with earlier delivery among term births.

## Abstract 2

Supplemental Digital Content is available in the text.

Short-term increases in aeroallergens have been associated with allergic and asthma exacerbations in the general population,^1–6^ and among children.^7,8^ In addition, one study found that exposure to high levels of pollen during late pregnancy was associated with an increased risk of hospitalization for asthma within the first year of life of children.^9^ Concerns have also been raised that exposure to high levels of aeroallergens during late pregnancy could act as a stimulus in triggering labor through an inflammatory process resulting from an abnormal production or early activation of cytokines that could result in earlier delivery.^9,10^ Therefore, if exposure to high levels of aeroallergens triggers labor in pregnant women (i.e., in both term and preterm pregnancies), the risk of delivery would be increased following days of high concentrations of aeroallergens. However, no study has investigated this issue specifically.

Fluctuation of aeroallergens has been closely related to climate, with increasing levels as warm seasons are expected to last longer. Although a number of studies have evaluated exposure to ambient temperature during pregnancy and the risk of earlier delivery, less is known about the potential impact of increasing levels of aeroallergens on pregnancy outcomes. Most studies showed an effect of exposure to high ambient temperature during the few days preceding delivery as possible factors affecting gestational length.^11–15^ Other studies found associations between warm season of births and reduced gestational length.^16,17^ However, these studies did not include measures of aeroallergens as possible factors affecting the duration of pregnancy.

Little is known regarding exposure to aeroallergens during pregnancy and global climate change will likely increase aeroallergen concentrations, such as pollen and fungal spores.^18–20^ Thus, this study aimed to evaluate the association between aeroallergen exposures during late pregnancy and the risk of early delivery among preterm and term births.

## METHODS

### Study Population and Design

We used the provincial birth record system BORN Ontario (Better Outcomes Registry & Network available at http://www.bornontario.ca) to extract all singleton births occurring in six cities where an aeroallergen monitor was available (i.e., Ottawa, Toronto, Hamilton, London, Windsor, Thunder Bay) in the province of Ontario, Canada, from April 1, 2004 to October 31, 2011. This real time, internet-based system captures hospital births and home births in the province of Ontario and contains information on maternal and prenatal characteristics, health services, intrapartum interventions, and maternal and infant outcomes. Between 2004 and 2011, data capture for all births across the province of Ontario improved from an estimated 82% to 100% of births. In fact, a quality assurance project conducted in 2008 showed that 96% of all births in Ontario were captured in the birth registry.^21^ Missing births were due to a small number of hospitals and midwifery practices, spread across the province, that were not participating in the birth registry in the early years.

A time-to-event study design was used by including all fetuses at risk during the time period under study. This approach has been previously suggested to account for potential biases due to short-term variation in seasonal patterns of conception that cannot be accounted for in time series or case-crossover studies.^11,17,22,23^ For each year, the risk period started on April 1st and ended on October 31st, which is the primary period for aeroallergens to be released in Canada. Births occurring during the cooler months when no aeroallergens are observed in Canada (i.e., November 1st to March 31st) were not considered events, but were included as fetuses at risk if they were at risk of preterm or term delivery during the risk period. This approach has been previously used in an article on maternal exposure to extreme heat for which the risk period, similar to ours, was limited to the summer months only.^11^ In addition, we had no information on birth outcomes for some women who were pregnant at the same time as participants of our study, but gave birth before the study started or after the study ended. To account for the noninclusion of these women, which has been described previously as the “fixed cohort bias,”^24,25^ we included only births with estimated conception dates ranging from 19 weeks (i.e., shortest pregnancy) before the study started to 44 weeks (i.e., longest pregnancy) before it ended. The final sample consisted of 225,234 births plus an additional 145,762 fetuses born after October, but at risk during April to October. We evaluated the risk of early delivery among preterm and term births based on prior literature that used a similar analysis approach.^11,25^ Specifically, we investigated whether exposure to aeroallergens during the week before birth was a trigger of early delivery resulting in preterm birth or resulting in early-term birth for those at term. Preterm delivery was defined as gestational age < 37 weeks, early term as 37–38 weeks, and full term as ≥39 weeks of gestation. Gestational age was determined from the mother’s last menstrual period, so there is potential for misclassification of gestational age, but there is no reason to suspect that misclassification differed by aeroallergen levels.

Ethics approval for this study was granted by the Health Canada and the Public Health Agency of Canada’s (PHAC) Research Ethics Board, the Children’s Hospital of Eastern Ontario and the Ottawa Health Science Network Research Ethics Board.

### Exposure Assessment

We obtained daily airborne allergens data from April to October of each year from Aerobiology Research Laboratories, which operates one aeroallergen monitoring station in each city under investigation. The original purpose of starting aeroallergen monitoring was due to the demand by allergists and the public for this kind of information across Canada. For each city, samplers are located in neighborhoods that have been identified by Aerobiology Research Laboratories to be representative of the biota in a 50-mile radius and are at about 6–7 feet from the ground, which is assumed to be representative of the air that people are breathing. The vegetation surrounding the local collecting station will have an impact on the pollen and fungal spore levels. However, the mixing of the air in the atmosphere does minimize this to a degree. Therefore, all samplers are located away from structures and as far away from the local trees as possible. This is to ensure the air can freely mix the aeroallergens that are present and make the collections more representative. At each station, rotational impaction methods were used to obtain 24-hour averages of aeroallergens (i.e., total pollen, grass pollen, tree pollen, weed pollen, and fungal spores). A description of the dominant species/taxa for each type of aeroallergens is provided in eTable 1 (http://links.lww.com/EDE/B120). The number of airborne allergen particles per cubic meter (m^3^) of air sample was estimated by analyzing the particles adhering to the silicon grease-coated sample rods. Aeroallergens are expressed as the total counts of pollen grains per m^3^ and the total number of spores per m^3^. Technicians doing the measurements for aeroallergens are trained palynologists and quality control between technicians is done throughout the year. Exposure to aeroallergens during pregnancy was defined as the total counts of pollen grains or spores on the day of birth or any of the six preceding days (i.e., the week before birth). We restricted the exposure window to 1 week because there is little evidence that earlier exposures could directly trigger labor via an inflammatory process. To improve accuracy and precision of exposure measurement, we included only pregnant women whose residence was within 20 km of the closest monitoring station, which is consistent with prior literature assessing the health impacts of exposure to aeroallergens across cities.^2,4^ We also conducted a sensitivity analysis that included only pregnant women whose residence was within 5 km of the closest monitoring station. There were no changes in the location of the monitoring stations during the study period.

### Covariates

Maternal characteristics considered potential confounders in this study were available from the BORN database and included maternal age at delivery, smoking during pregnancy, parity, previous preterm delivery, previous caesarean section delivery, and status of any of the following maternal medical comorbidities (chronic asthma, chronic hypertension, gestational diabetes, prepregnancy type I and II diabetes mellitus). Because we did not have any individual-level socioeconomic status (SES) variables, we evaluated confounding SES by extracting three SES variables from the 2006 Canada Census at the dissemination-area (DA) level: median family income; proportion of population in the DA who are visible minority; and percentage of the adult female population who were 25–64 years old with a university degree. A dissemination area is a small geographical unit composed of one or more neighboring dissemination blocks, with a population of 400 to 700 persons. All of Canada is divided into dissemination areas. These area-based variables have been shown to be reasonable measures of neighborhood-level SES.^26^ We assigned the three SES variables to study subjects based on their postal codes at the time of delivery. No issues of collinearity between the three SES variables were observed in this study.

Daily mean temperature, relative humidity, and barometric pressure, considered potential confounders in the current analysis, were provided by Environment Canada using the average of measurements of all weather stations within each city. Daily average measurements of ambient air pollutants PM_2.5_, NO_2_, and O_3_, which are also potential confounders, were obtained for each city through automated fixed-site monitoring stations from the National Air Pollution Surveillance (NAPS) network provided by Environment Canada. There were two air pollution monitoring stations in Ottawa, 12 in Toronto, two in Hamilton, two in London, two in Windsor, and one in Thunder Bay. Both meteorologic and air pollution factors were measured continuously and were captured for the week preceding birth based on previous research.^11,25^ We also included a time-dependent categorical variable for month/year of follow-up (e.g., April 2004, May 2004, etc.) to account for seasonality in the analysis. This approach is similar to a previous article investigating maternal exposure to aeroallergens, which adjusted for year and season of birth.^9^ In addition, a previous study evaluating effects of ambient heat on early delivery using a time-to-event approach used a similar adjustment to ours for seasonality and long-term trends.^11^ In addition, we included a natural cubic spline with four degrees of freedom for day of the year to remove residual confounding due to within-month variation.

### Statistical Analysis

Our analysis consisted of two stages. In the first stage, we applied Cox proportional hazards models separately in each city to investigate the risk of early delivery in three gestational periods (i.e., preterm, early-term, and full-term periods) using gestational age in days as the time scale.^27^ We obtained city-specific hazard ratios (HRs) with their 95% confidence intervals (CIs) to evaluate the effect of short-term exposure to aeroallergens during pregnancy on the risk of early delivery. For each gestational period, we calculated the HR associated with changes in aeroallergens on the probability of giving birth (compared with remaining pregnant). Similar to a previous study evaluating the relationship between high ambient temperature and risk of early delivery, we applied different censoring approaches depending on the gestational period.^11^ Specifically, when investigating the risk of early delivery during the preterm period, we censored pregnancies >37 weeks of gestation. For the risk of early delivery during the early-term period, we censored pregnancies ≥39 weeks of gestation and excluded births <37 weeks. We also evaluated whether aeroallergens could trigger delivery among full-term births and for this analysis we excluded births <39 weeks of gestation. In particular, a HR above one among preterm pregnancies represents an increased risk of preterm birth associated with changes in aeroallergens, while a HR above one during the early-term period represents an increased risk of early-term birth due to changes in aeroallergens. Finally, a hazard ratio above one among full-term pregnancies corresponds to an increased risk of early delivery due to the effect of aeroallergens. For each gestational period, we also censored pregnancies that delivered during November to March that were part of the pool of fetuses at risk from April to October.

For each location, we specified the aeroallergens-associated risk of early delivery relationship with a distributed lag linear model.^28^ We accounted for the potential lagged effects, as well as for the potential nonlinear effect of daily mean ambient temperature, the linear effect of relative humidity and the linear effect of ambient air pollution on the hazard of birth using the distributed lag nonlinear methodology,^27^ A 6-day lag period was specified to evaluate the lagged effect of all environmental measures on the hazard of birth. All environmental factors were available as time-dependent variables for each day of gestation starting at week 19 until birth. The relationship between aeroallergens and the risk of early delivery is presented for an interquartile range width (IQR_*w*_) increase for each type of aeroallergen. We also modeled aeroallergens in categories based on quintiles, as did previous work investigating aeroallergens as an exposure.^2^ The lagged effect of aeroallergens was modeled with a natural cubic spline with an intercept and two internal knots placed at equally spaced values in the log scale. This represents the temporal change in risk after a specific exposure, and it estimates the distribution of immediate and delayed effects that cumulate across the lag period. We fitted the potential nonlinear effect of daily mean temperature over a week period on the hazard of birth using a natural cubic B-spline with three internal knots placed at the 10th, 50th, and 90th percentiles of city-specific temperature distributions.^11^ Relative humidity and air pollution variables were also evaluated on a continuous basis over a week period. The Akaike information criterion and Bayesian information criterion adapted to survival analysis were used for optimal model selection.^27^ Finally, in a second stage, we pooled the estimated city-specific overall aeroallergens-hazard of birth associations using a multivariate meta-analytical model.^29,30^

We evaluated potential confounders such as markers of air pollution (PM_2.5_, NO_2_, and O_3_), weather variables, neighborhood-level factors, and maternal characteristics in the multivariable models using a backward deletion approach.^31^ This was accomplished by adjusting for all potential confounders and then removing the least significant confounding variables one by one in a stepwise manner as long as the total proportional change in the estimate compared with the fully adjusted model was less than 10%. We tested the proportional hazards assumption using aeroallergens-by-gestational age interaction terms. Analyses were performed with the R software, version 3.1.3, using packages *dlnm*, version 2.1.4, and *mvmeta*, version 0.4.5.

## RESULTS

Among a total of 225,234 births, there were 14,064 preterm births, 56,897 early-term births, and 154,273 full-term births (Table 1). About 56% of all births occurred in the city of Toronto (n = 125,294; eTable 2; http://links.lww.com/EDE/B120). Among them, 9% reported smoking during pregnancy, 8% had medical comorbidities, and the majority (55%) previously given birth. A total of 24,776 women were pregnant more than once over the period under investigation (data not shown). During the study period, total pollen concentrations ranged from 0 to 5,002.0 grains/m^3^ and total fungal spores from 0 to 108,241.1 counts/m^3^ (Table 2). Total pollen concentrations reached their highest levels in the city of Ottawa, while the city of Thunder Bay had the highest levels of fungal spores (eTable 3; http://links.lww.com/EDE/B120). Weak correlations were observed between aeroallergens variables, except between total pollen and tree pollen (Pearson correlation coefficient = 0.98; eTable 4; http://links.lww.com/EDE/B120). Less than 5% of days throughout the study period had missing information on aeroallergens.

**TABLE 1.**
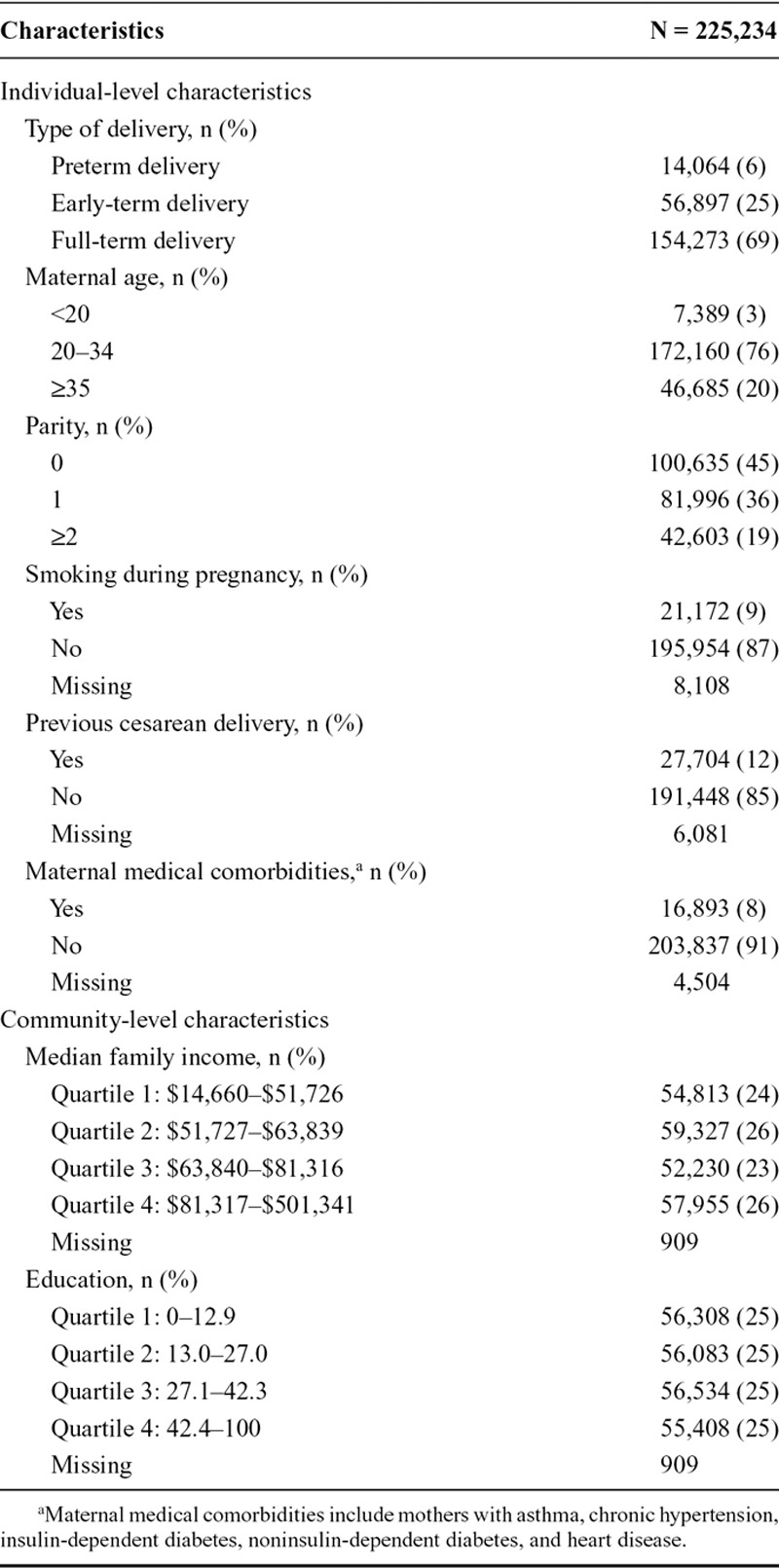
Demographic and Pregnancy-related Characteristics of Study Participants, Ontario, Canada, April–October, 2004–2011

**TABLE 2.**
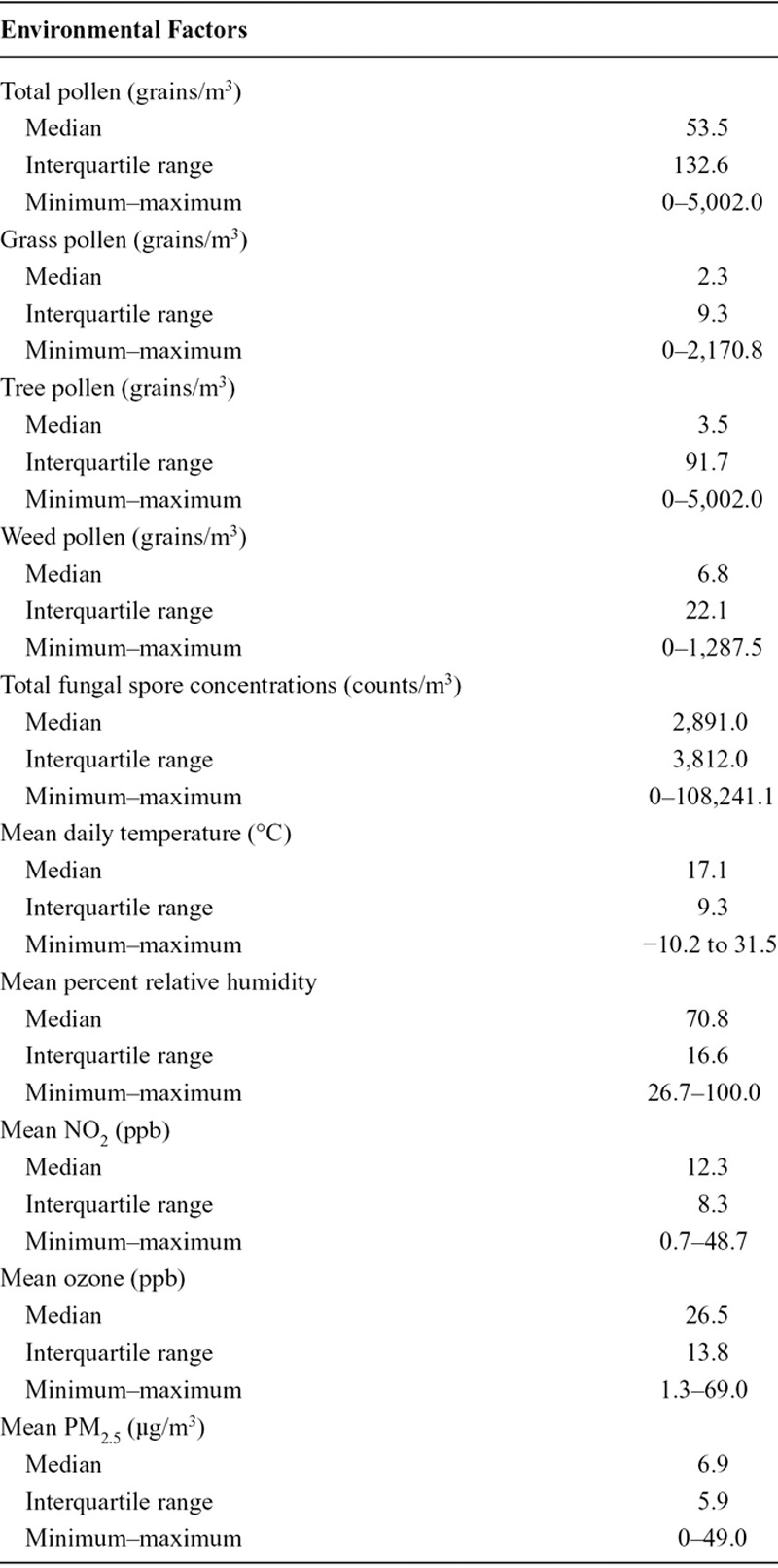
Environmental Factors During the Week Preceding Birth, Ontario, Canada, April–October, 2004–2011

**FIGURE 1.**
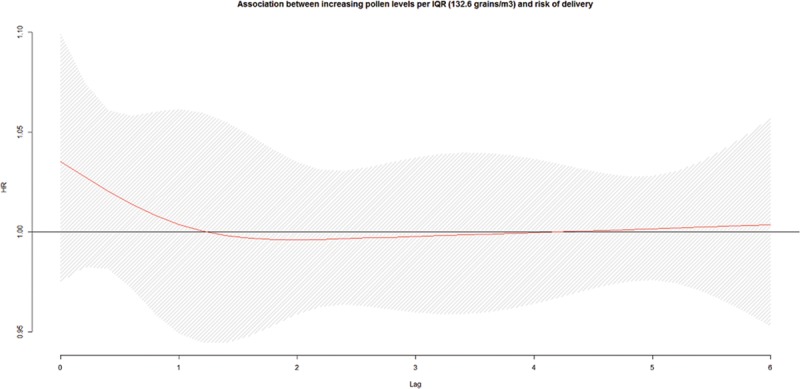
Association^a,b^ between ambient total pollen concentrations during the preceding week and risk of delivery among preterm pregnancies, per IQR width (132.6 grains/m^3^), pooled across six cities in Ontario, Canada, April–October, 2004–2011. a: Hazard ratios adjusted for maternal age at delivery, parity, smoking during pregnancy, dissemination-area median family income, percentage of the dissemination-area adult female population 25–64 years with a university degree, proportion of dissemination-area population who are visible minority, month/year of follow-up, ambient temperature, relative humidity, NO_2_, and O_3_. b: The hazard ratios are shown as a smooth black line (red in the online version) and the pointwise 95% confidence intervals are shown in the shaded area.

**FIGURE 2.**
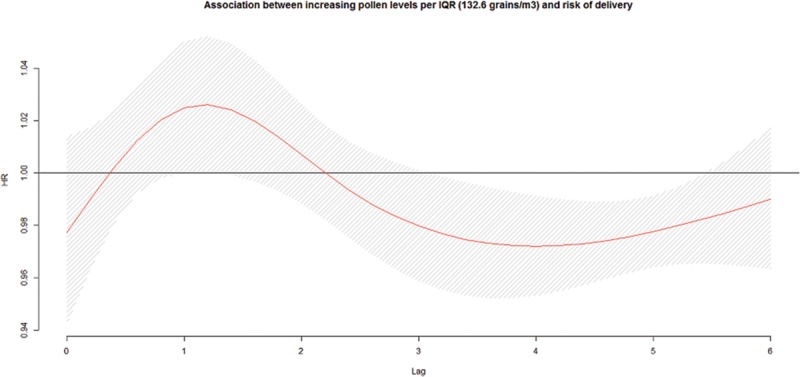
Association^a,b^ between increasing ambient total pollen concentrations per IQR width (132.6 grains/m^3^) during the preceding week and risk of delivery among early-term pregnancies, pooled across six cities in Ontario, Canada, April–October, 2004–2011. a. Hazard ratios adjusted for maternal age at delivery, parity, smoking during pregnancy, dissemination-area median family income, percentage of the dissemination-area adult female population 25–64 years with a university degree, proportion of dissemination-area population who are visible minority, month/year of follow-up, ambient temperature, relative humidity, NO_2_, and O_3_. b: The hazard ratios are shown as a smooth black line (red in the online version) and the pointwise 95% confidence intervals are shown in the shaded area.

**FIGURE 3.**
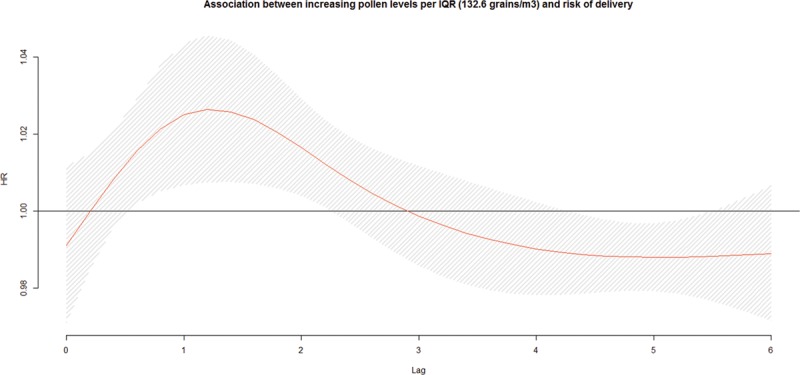
Association^a,b^ between increasing ambient total pollen concentrations per IQR width (132.6 grains/m^3^) during the preceding week and risk of delivery among full-term pregnancies, pooled across six cities in Ontario, Canada, April–October, 2004–2011. a: Hazard ratios adjusted for maternal age at delivery, parity, smoking during pregnancy, dissemination-area median family income, percentage of the dissemination-area adult female population 25–64 years with a university degree, proportion of dissemination-area population who are visible minority, month/year of follow-up, ambient temperature, relative humidity, NO_2_, and O_3_. b: The hazard ratios are shown as a smooth black line (red in the online version) and the pointwise 95% confidence intervals are shown in the shaded area.

We observed no association between increasing total pollen concentrations during the preceding week and the risk of early delivery among preterm pregnancies (Figure 1). We observed an association with early-term births at lag 1 (HR = 1.02; 95% CI: 1.00, 1.04 per IQR, 132.6; Figure 2). We also investigated longer lag periods (i.e., over a week), but most associations were imprecise (eTable 5 in online Appendix; http://links.lww.com/EDE/B120). Similar results were observed among full-term births, with increased HRs at lags 1 (HR = 1.03; 95% CI: 1.01, 1.05) and 2 (HR = 1.02; 95% CI: 1.01, 1.03; Table 3 and Figure 3).

**TABLE 3.**
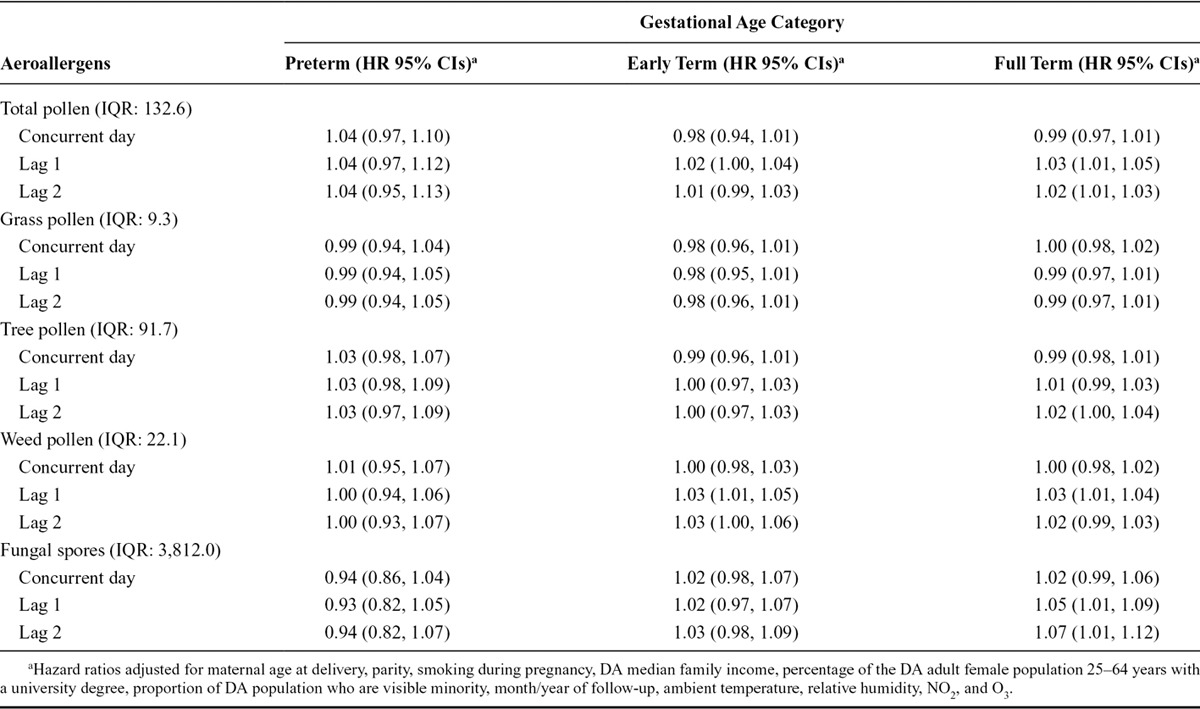
Associations Between Exposure to Aeroallergens and the Risk of Delivery Across Lagged Days Among Preterm, Early- and Full-term Pregnancies, Pooled Across Six Cities in Ontario, Canada, April–October, 2004–2011

Lagged associations between each subtype of aeroallergens and the risk of early delivery among preterm, early-term, and full-term births are presented in Table 3. Increasing levels of weed pollen at lag 1 day were associated with a 3% higher risk of early delivery among early-term births (HR = 1.03; 95% CI: 1.01, 1.05) and among full-term births (HR = 1.03; 95% CI: 1.01, 1.04) per IQR_*w*_ (22.1 grains/m^3^). In addition, increasing levels of fungal spores were associated with an increased risk of early delivery among full-term births at lags 1 (HR = 1.05; 95% CI: 1.01, 1.09) and 2 (HR = 1.07; 95% CI: 1.01, 1.12) per IQR_*w*_ (3,812.0 counts/m^3^). No associations were observed among preterm deliveries.

**TABLE 4.**
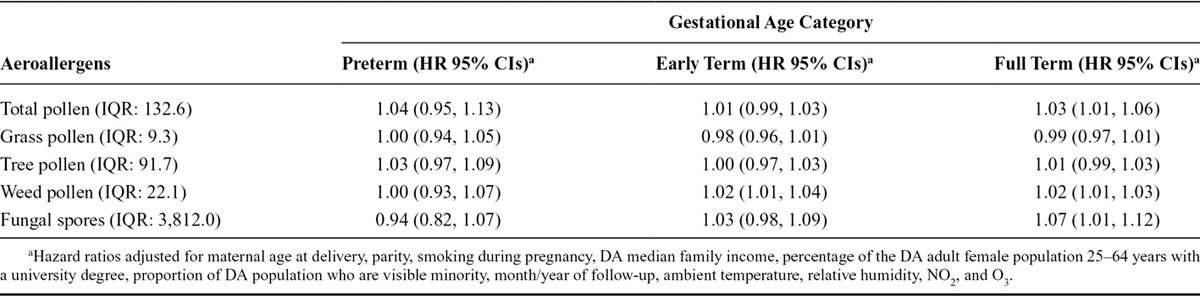
Cumulative Effect Over Lag 0 to 2 Days from Exposure to Aeroallergens on the Risk of Delivery Among Preterm, Early- and Full-term Pregnancies, Pooled Across Six cities in Ontario, Canada, April–October, 2004–2011

The cumulated estimated effect over lags 0 to 2 days showed that weed pollen was associated with increased risk of early delivery by 2% for every IQR_*w*_ (22.1) increase among early-term (HR = 1.02; 95% CI: 1.01, 1.03) and full-term (HR = 1.02; 95% CI: 1.01, 1.03) pregnancies. Total pollen (HR = 1.03, 95% CI: 1.01, 1.06) and fungal spores (HR = 1.07; 95% CI: 1.01, 1.12) also were associated with increased risk of early delivery among full-term pregnancies when the effect was cumulated over 0 to 2 days. When restricting to births within 5 km of the monitors (eTable 6; http://links.lww.com/EDE/B120), all associations imprecise, but were all stronger in magnitude. Additional analyses based on quintile categories of aeroallergens showed that the highest categories (Q5) of exposure to total pollen and weed pollen were associated with early delivery among early-term births (Table 4, eTable 7: http://links.lww.com/EDE/B120). For all overall effects calculated, there was no heterogeneity across cities. For instance, eFigure 1 (http://links.lww.com/EDE/B120) shows that estimated effects of weed pollen cumulated over lag 0 to 2 days among early-term pregnancies were similar in magnitude across each city (*I*^2^ = 0.0; *P* = 0.99). Aeroallergen-by-gestational-age interaction terms suggested that hazards were proportional over the preterm, early-term, and full-term periods.

## DISCUSSION

In this study, we investigated the risk of early delivery in association with increasing airborne pollen and fungal spores among a retrospectively created profile of exposure among pregnant women living in six large cities in the province of Ontario, Canada. We found that increasing levels of weed pollen were associated with increased risk of delivery among early- and full-term pregnancies and that total pollen and fungal spores were positively associated with the risk of delivery among full-term pregnancies. Findings of this study suggest that exposure to aeroallergens may shorten gestational length of pregnancies that reach term during the pollen season, but not enough to discernibly affect preterm birth rates. This study may have implications in reducing risks of early-term births as evidence is emerging regarding increased risk of childhood morbidity and mortality among those born early term compared with those born later term.^32–34^

To our knowledge, this is the first study to investigate the association between specific types of aeroallergens and the risk of delivery. Previous studies have investigated mostly the association between season of birth, including pollen season, and birth outcomes. The relationship between season of birth and gestational length has been examined in a recent review.^16^ Authors found that in studies conducted in the northern hemisphere, there was a relationship between season of birth and rate of preterm birth with a peak in January and another in June.^16^ However, the evaluation of the seasonality of birth is limited by the difficulty of disentangling the potential factors driving the risk of delivery. In addition, analyses based on seasonality of birth neglect that it is fetuses that are at risk and not only newborns. For instance, the annual pattern of birth may be affected by seasonal preferences in pregnancy planning or increases in coital frequency during holidays, which can affect the seasonal variation in the numbers of fetuses at risk of delivery.^17^ Therefore, it is difficult to directly compare this with previous studies investigating seasonality of birth.

The underlying mechanism by which airborne pollen and fungal spores may affect the risk of earlier delivery is still uncertain. One possible mechanism could be related to an immune response triggering labor following exposure to short-term fluctuations in pollen. During pregnancy, the fetus develops within the mother without succumbing to immunological rejection because of the establishment of a fetomaternal tolerance.^35^ The latter is characterized by a balance between innate and adaptive immune cells between the fetus and the mother creating an anti-inflammatory environment which sustains pregnancy.^10^ Labor occurs when an inflammatory response is initiated through the secretion of cytokines/chemokines infiltrating reproductive tissues to create a proinflammatory microenvironment, which leads to delivery.^36,37^ It is likely that pregnant women sensitive to elevated levels of airborne pollen and/or fungal spores may suffer from allergy symptoms. Airborne pollen and fungal spores could act as a triggered stimulus of the immune system that could induce an earlier activation of cytokines/chemokines infiltrating reproductive tissues, eliciting a shift from an anti-inflammatory to a proinflammatory microenvironment and consequently earlier labor among term pregnancies. In fact, season of birth has been associated with a range of differences in cord blood cytokine response profiles, which may support our hypothesis.^38^

Weed pollen was the only type of pollen associated with an increased risk of delivery among early- and full-term pregnancies. In fact, weed pollen is the most important cause of allergic rhinitis in the north-east part of North America and is likely responsible for 50% to 90% of seasonal allergies in this area.^39^ Thus, our findings may reflect an adverse immune response among pregnant women who have seasonal allergies to weed pollen that triggers labor late in gestation. Consequently, women who have allergic responses to weed pollen may be more vulnerable to an adverse reaction to aeroallergens and consequently earlier delivery during pregnancy. However, in this study, we did not have information on allergy history and we could not stratify analyses by allergy status.

In this study, weed pollen was associated with the risk of delivery among early-term pregnancies. The health impacts of children born early term are still not clear, but some evidence is emerging regarding long-term health impacts. For instance, a national cohort study in Sweden showed increased risk of mortality in infancy, early childhood, and young adulthood among early-term births compared with later-term births.^32^ Another study showed increased risk of hospital admissions during childhood among infants born early term compared with those born later term.^33^ Thus, the triggering of labor by weed pollen resulting in early-term birth could carry lifelong consequences.

This study has some limitations. First, we relied on fixed-site regional monitors to estimate personal exposures to ambient aeroallergens, weather variables, and markers of air pollution which may have resulted in exposure misclassification due to spatial heterogeneity. However, this misclassification was likely nondifferential and thus likely led to an underestimation of the effects. For instance, effect estimates, although imprecisely measured, were stronger when restricting analysis to births (N = 19,174) within 5 km from an aeroallergens monitoring station. Second, while we included several important confounding factors, we cannot rule out potential residual confounding by unmeasured factors, such as individual ethnicity or socioeconomic status information. Third, we did not have information on pollen allergy status among pregnant women as those women might be more vulnerable to the effects of pollen on risk of earlier delivery. This is an important avenue for future research to investigate the potential modifying effect of this condition in the associations under investigation. Notable strengths of this study include the large sample size, the use of a distributed lag linear model in a time-to-event analysis to characterize fully the exposure–response relationship, the range of maternal risk factors to account for potential confounding, and the availability of ambient aeroallergens data across several regions.

This study showed that increasing levels of ambient weed pollen were associated with risk of delivery among early- and full-term pregnancies and that increasing levels of fungal spores were associated with risk of delivery only among full-term pregnancies. If shown to be causal, these results suggest that certain types of ambient aeroallergens could trigger labor late in gestation. Future studies should be conducted to verify these findings.

## ACKNOWLEDGMENTS

The authors thank Dr. Ann Sprague and Daniel Bedard for facilitating access to the BORN database.

## Supplementary Material
