# Supplementary material for: Maternal Exposure to Aeroallergens and the Risk of Early Delivery
Source: Epidemiology. 2016 Nov 30;28(1):107–15. doi: 10.1097/EDE.0000000000000573 (PMC5380107; doi:10.1097/EDE.0000000000000573)
Supplement: Supplementary file 1 [file ede-28-107-s001.pdf]

eTable 1. Dominant species/taxa for each type of aeroallergens.

Aeroallergens	Dominant species/taxa
Weed pollen	AMBROSIA (Ragweed)<br>ARTEMISIA (Sagebrush, Wormwood)<br>CHENOPODIACEAE, AMARANTHACEAE<br>CRUCIFERAE (Mustards)<br>HUMULUS, CANNABIS (Hops, Hemp)<br>LYTHRUM (Purple Loostrife)<br>Misc. Compositeae<br>Misc. Weeds<br>PLANTAGO (Plantain)<br>RUMEX (Dock, Rhubarb, Sorrel)<br>SALSOLA PESTIFER (Russian Thistle)<br>SOLIDAGO (Goldenrod)<br>TYPHA (Cattail)<br>UMBELLIFERAE (Wild Carrot)<br>URTICACEAE (Nettles & Pellitory)
Tree pollen	CUPRESSACEAE (Cedar, Cypress, Juniper, Thuja)<br>LARIX (Larch, Tamarak, Pseudotsuga)<br>PINACEAE (Pine, Fir, Spruce)<br>TSUGA CANADENSIS/TSUGA HETEROPHYLLA (Hemlock)<br>ACER (Boxelder, Maple)<br>AESCULUS (Buckeye, Horse Chestnut)<br>ALNUS (Alder)<br>BETULA (Birch)<br>Birch look-a-likes (Hornbeam, Hop-Hornbeam)<br>CARYA (Hickory)<br>CASTANEA (Chestnut)<br>CELTIS (Hackberry)<br>CORYLUS (Hazelnut)<br>FAGUS (Beech)<br>FRAXINUS (Ash)<br>JUGLANS (Walnut)

	MORUS (Mulberry)<br>OLEACEAE (Ligustrum, Syringa)<br>PLATANUS (Sycamore)<br>POPULUS (Aspen, Poplar)<br>PRUNUS, MALUS, CRATAEGUS (Apple, Plum, Pear, Hawthorn)<br>QUERCUS (Oak)<br>SALIX (Willow)<br>SAMBUCUS (Elderberry)<br>TILIA (Basswood, Linden)<br>ULMUS (Elm)
Grass pollen	CYPERACEAE (Sedge family)<br>GRAMINEAE (True Grasses)
Spores	ASCOMYCETES<br>BASIDIOMYCETES<br>FUNGI IMPERFECTI<br>MYXOMYCETES<br>ZYGOMYCETES

eTable 2. Number of births by city, Ontario, Canada, April-October 2004-2011.

Location	Number of births
All Sites Combined	225,234
Toronto	125,294
Hamilton	36,634
London	25,068
Ottawa	16,020
Thunder Bay	4,839
Windsor	17,379

eTable 3. Descriptive statistics of aeroallergens across each city during the week preceding birth, Ontario, Canada, April-October 2004-2011.

Aeroallergens	Toronto	Hamilton	London	Ottawa	Thunder Bay	Windsor
Total pollen (grains/m^3)						
Median	55.9	51.4	51.2	71.4	30.3	68.3
Interquartile range	126.0	188.7	160.6	171.1	103.3	157.5
Minimum-maximum	0 – 3611.5	0 – 1780.4	0 – 1780.4	0 – 5002.0	0 – 3805.9	0 – 3991.3
Grass pollen (grains/m^3)						
Median	2.3	2.2	2.3	1.2	0.8	3.1
Interquartile range	9.3	8.6	9.1	8.3	4.5	11.7
Minimum-maximum	0 – 184.1	0 – 355.2	0 – 355.2	0 – 977	0 – 2170.8	0 – 319.4
Tree pollen (grains/m^3)						
Median	3.1	4.7	2.6	5.6	2.4	3.5
Interquartile range	88.8	184.2	139.1	148.6	79.4	88.9
Minimum-maximum	0 – 3611.5	0 – 1780.4	0 – 1780.4	0 – 5002.0	0 – 3805.9	0 – 3991.3
Weed pollen (grains/m3)						
Median	7.5	4.4	5.9	5.9	2.0	11.0
Interquartile range	22.3	16.4	19.4	31.8	9.0	33.9
Minimum-maximum	0 – 755.3	0 – 319.6	0 – 381.8	0 – 613.6	0 – 210.9	0 – 1287.5
Total spore concentrations (counts/m^3)						
Median	2661.7	2560.9	2754.8	3843.5	4524.0	2679.8
Interquartile range	3462.7	3,947	3612.0	4,953.1	6890.0	3422.8
Minimum-maximum	0 – 47,424.9	11.1 – 27,870.5	11.1 – 27,870.5	0 – 30,469.9	3.7 – 108,241.1	18.7 – 39,265.2

eTable 4. Pearson correlation coefficients between aeroallergens variables, Ontario, Canada, April-October 2004-2011.

	Pollen	Weeds	Grasses	Trees	Spores
Pollen	1	0.01	0.05	0.98	- 0.17
Weeds		1	- 0.04	- 0.17	0.25
Grasses			1	-0.03	0.09
Trees				1	- 0.22
Spores					1

eTable 5. Associations between exposure to aeroallergens and the risk of delivery across lagged days among preterm, early term and full term pregnancies, pooled across 6 cities in Ontario, Canada, April–October, 2004-2011.

**Aeroallergens**	**Gestational age category**		
	**Preterm (HR 95% CIs)[1]**	**Early term (HR 95% CIs)[1]**	**Full term (HR 95% CIs)[1]**
Total pollen (IQR: 132.6)			
Lag 3	0.99 (0.96, 1.03)	0.98 (0.96, 1.00)	1.00 (0.99, 1.01)
Lag 4	1.00 (0.97, 1.03)	0.97 (0.96, 0.99)	0.99 (0.98, 1.00)
Lag 5	1.00 (0.98, 1.03)	0.98 (0.97, 0.99)	0.99 (0.98, 0.99)
Lag 6	1.01 (0.95, 1.06)	0.99 (0.97, 1.02)	0.99 (0.97, 1.01)
Grass pollen (IQR: 9.3)			
Lag 3	0.99 (0.94, 1.05)	0.98 (0.96, 1.01)	0.99 (0.97, 1.01)
Lag 4	0.99 (0.94, 1.05)	0.99 (0.97, 1.02)	0.99 (0.97, 1.01)
Lag 5	0.99 (0.94, 1.05)	0.98 (0.96, 1.01)	0.99 (0.97, 1.01)
Lag 6	0.99 (0.94, 1.05)	0.98 (0.96, 1.01)	0.99 (0.97, 1.01)
Tree pollen (IQR: 91.7)			
Lag 3	1.02 (0.98, 1.07)	1.00 (0.97, 1.03)	1.01 (0.99, 1.02)
Lag 4	1.01 (0.97, 1.06)	1.00 (0.97, 1.03)	1.00 (0.98, 1.03)
Lag 5	1.00 (0.95, 1.06)	1.00 (0.98, 1.03)	1.00 (0.98, 1.02)
Lag 6	1.00 (0.95, 1.06)	1.00 (0.98, 1.03)	1.00 (0.98, 1.02)
Weed pollen (IQR: 22.1)			
Lag 3	1.00 (0.95, 1.06)	1.02 (1.00, 1.04)	1.02 (0.98, 1.02)
Lag 4	1.00 (0.94, 1.07)	1.02 (0.99, 1.04)	1.01 (0.98, 1.02)
Lag 5	1.00 (0.95, 1.06)	1.01 (0.99, 1.03)	1.00 (0.97, 1.02)
Lag 6	1.00 (0.95, 1.06)	0.99 (0.97, 1.03)	1.00 (0.97, 1.03)
Spores (IQR: 3812.0)			
Lag 3	0.94 (0.82, 1.07)	1.02 (0.97, 1.09)	1.07 (1.00, 1.11)
Lag 4	0.95 (0.81, 1.08)	1.02 (0.97, 1.09)	1.03 (0.98, 1.08)
Lag 5	0.95 (0.81, 1.09)	1.01 (0.96, 1.09)	1.02 (0.98, 1.07)
Lag 6	0.95 (0.81, 1.09)	1.00 (0.96, 1.10)	1.02 (0.97, 1.06)

1. Hazard ratios adjusted for maternal age at delivery, parity, smoking during pregnancy, dissemination-area (DA) median family income, percentage of the DA adult female population 25-64 years with a university degree, proportion of DA population who are visible minority, month/year of follow-up, ambient temperature, relative humidity, NO_2 and O_3.

eTable 6. Cumulative effect over lag 0 to 2 days from exposure to aeroallergens on the risk of delivery among preterm, early term and full term pregnancies (N = 39,174), limited to a 5km buffer from aeroallergens stations, pooled across 6 cities in Ontario, Canada, April–October, 2004-2011.

Aeroallergens	Gestational age category		
	**Preterm (HR 95% CIs)[1]**	**Early term (HR 95% CIs)[1]**	**Full term (HR 95% CIs)[1]**
Total pollen (IQR: 132.6)	1.06 (0.95, 1.14)	1.10 (0.94, 1.30)	1.14 (0.93, 1.39)
Grass pollen (IQR: 9.3)	1.00 (0.92, 1.07)	0.99 (0.97, 1.01)	1.00 (0.97, 1.01)
Tree pollen (IQR: 91.7)	1.04 (0.96, 1.10)	1.07 (0.95, 1.12)	1.10 (0.94, 1.30)
Weed pollen (IQR: 22.1)	1.01 (0.92, 1.09)	1.12 (0.95, 1.37)	1.13 (0.94, 1.36)
Fungal spores (IQR: 3812.0)	0.98 (0.81, 1.09)	1.14 (0.93, 1.38)	1.17 (0.91, 1.41)

1. Hazard ratios adjusted for maternal age at delivery, parity, smoking during pregnancy, dissemination-area (DA) median family income, percentage of the DA adult female population 25-64 years with a university degree, proportion of DA population who are visible minority, month/year of follow-up, ambient temperature, relative humidity, NO2 and O3.

eTable 7. Cumulative effect over lag 0 to 2 days from exposure to aeroallergens on the risk of delivery among preterm, early term and full term pregnancies, across quintiles for each aeroallergens, pooled across 6 cities in Ontario, Canada, April–October, 2004-2011.

	Gestational age category		
**Aeroallergens**	**Preterm (HR 95% CIs)[1]**	**Early term (HR 95% CIs)[1]**	**Full term (HR 95% CIs)[1]**
Total pollen			
Q1 (0 – 21.0)	1.0	1.0	1.0
Q2 (21.0 – 42.1)	0.96 (0.72 – 1.29)	1.07 (0.91 – 1.24)	0.99 (0.89, 1.09)
Q3 (42.1 – 96.7)	1.04 (0.64 – 1.70)	1.09 (0.87 – 1.38)	1.01 (0.88, 1.16)
Q4 (96.7 – 224.3)	0.80 (0.50 – 1.30)	1.21 (0.93 – 1.57)	1.02 (0.88, 1.17)
Q5 (224.3 – 5002.0)	1.23 (0.72 – 2.07)	1.28 (1.01 – 1.63)	1.22 (0.99, 1.52)
Grass pollen			
Q1 (0 – 0.3)	1.0	1.0	1.0
Q2 (0.3 – 1.5)	0.99 (0.88, 1.10)	0.98 (0.87, 1.11)	0.99 (0.85, 1.13)
Q3 (1.5 – 3.6)	1.00 (0.89, 1.11)	1.00 (0.88, 1.10)	1.01 (0.87, 1.12)
Q4 (3.6 – 15.4)	1.01 (0.88, 1.13)	0.99 (0.86, 1.13)	0.99 (0.88, 1.11)
Q5 (15.4 – 2170.8)	0.98 (0.87, 1.14)	0.99 (0.87, 1.11)	0.98 (0.86, 1.14)
Tree pollen			
Q1 (0 – 1.5)	1.0	1.0	1.0
Q2 (1.5 – 2.0)	1.01 (0.92, 1.16)	1.00 (0.93, 1.14)	1.00 (0.90, 1.15)
Q3 (2.0 – 9.4)	1.00 (0.90, 1.13)	1.01 (0.91, 1.12)	1.01 (0.92, 1.11)
Q4 (9.4 – 160.2)	1.01 (0.90, 1.14)	1.00 (0.92, 1.17)	0.99 (0.87, 1.19)
Q5 (160.2 – 5002.0)	0.98 (0.88, 1.14)	0.99 (0.86, 1.19)	0.99 (0.88, 1.17)
Weed pollen			
Q1 (0 – 1.7)	1.0	1.0	1.0
Q2 (1.7 – 5.4)	1.02 (0.80, 1.20)	1.06 (0.93, 1.20)	1.05 (0.97, 1.15)
Q3 (5.4 – 14.2)	1.04 (0.82, 1.19)	1.11 (0.88, 1.35)	1.13 (0.90, 1.33)
Q4 (14.2 – 34.2)	1.03 (0.90, 1.11)	1.20 (0.95, 1.50)	1.17 (0.98, 1.40)
Q5 (34.2 – 1287.5)	1.01 (0.78, 1.25)	1.34 (1.03, 1.70)	1.32 (1.02, 1.60)
Fungal spores			
Q1 (0 – 863.2)	1.0	1.0	1.0
Q2 (863.2 – 2330.2)	0.80 (0.42 – 1.53)	0.97 (0.68 – 1.37)	0.97 (0.79, 1.19)

Q3 (2330.2 – 3687.6)	0.73 (0.37 – 1.47)	1.14 (0.81 – 1.61)	1.16 (0.90, 1.31)
Q4 (3687.6 – 5327.0)	0.84 (0.42 – 1.68)	1.28 (0.95 – 1.72)	1.27 (0.95, 1.72)
Q5 (5327.0 – 108,241.1)	1.04 (0.57 – 1.90)	1.30 (0.94 – 1.82)	1.35 (1.02, 1.70)

1. Hazard ratios adjusted for maternal age at delivery, parity, smoking during pregnancy, dissemination-area (DA) median family income, percentage of the DA adult female population 25-64 years with a university degree, proportion of DA population who are visible minority, month/year of follow-up, ambient temperature, relative humidity, NO2 and O3.

eFigure 1. Associations[1] between ambient weed pollen concentrations cumulated over lag 0 to 2 days and risk of delivery among early term pregnancies, per IQR (22.1), for each city and pooled across 6 cities in Ontario, Canada, April–October, 2004-2011.

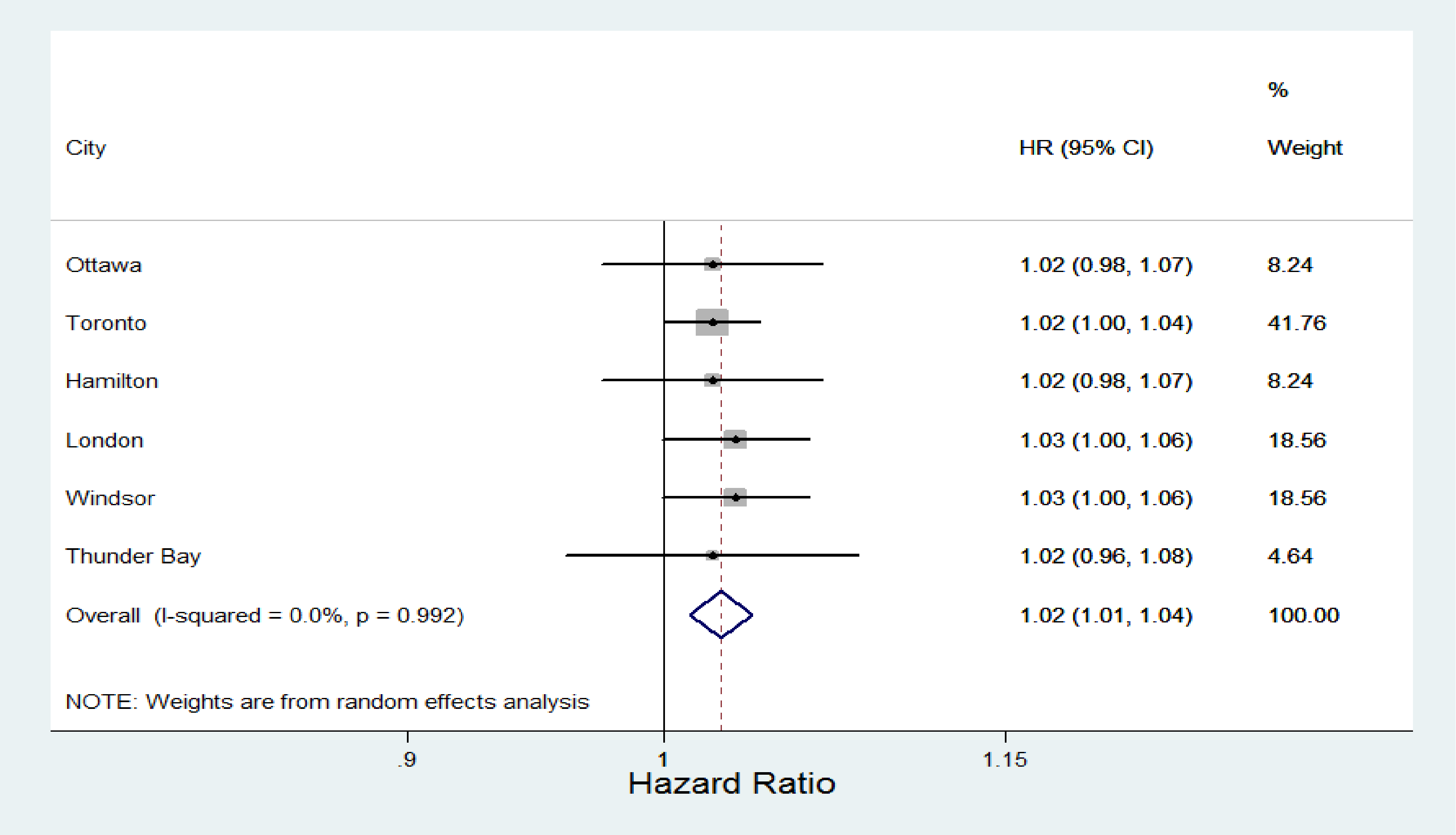

1. Hazard ratios adjusted for maternal age at delivery, parity, smoking during pregnancy, dissemination-area (DA) median family income, percentage of the DA adult female population 25-64 years with a university degree, proportion of DA population who are visible minority, month/year of follow-up, ambient temperature, relative humidity, NO_2 and O_3.
